# Chloroform in Tic Douloureux

**Published:** 1885-01

**Authors:** 


### Chloroform in Tic Douloureux.

Bartholow, in presenting a case of tic douloureux to his class, proceeds to make the following remarks on the treatment of this intractable disorder: "There is no fact in therapeutics more striking than the curative effects of a few drops of chloroform injected in the neighborhood of this division of the nerve (the superior maxillary branch of the fifth cranial nerve) when the seat of neuralgia. It is this division which is most frequently affected, and fortunately so, for it is this division which is most easily reached by the following method of treatment: Given a case of tic douloureux involving this nerve, lift the corner of the lip and insert a hypodermic needle at the junction of the mucous membrane of the lip and that of the cavity of the mouth, pass it up till its extremity comes in the neighborhood of the nerve and inject from five to fifteen minims of chloroform or ether, the former the most efficient. In the majority of cases there is immediate relief of pain, which, if not permanent, lasts a considerable time. I have a patient in Boston who comes to me twice a year to have this injection practiced. In his case no other measures have answered. The relief which he obtains is complete, and lasts never less than six months.

